# Carbohydrate composition, viscosity, solubility, and sensory acceptance of sweetpotato- and maize-based complementary foods

**DOI:** 10.3402/fnr.v57i0.18717

**Published:** 2013-03-15

**Authors:** Francis Kweku Amagloh, Anthony N. Mutukumira, Louise Brough, Janet L. Weber, Allan Hardacre, Jane Coad

**Affiliations:** 1 Institute of Food, Nutrition and Human Health, College of Health, Massey University, Palmerston North, New Zealand; 2 Department of Applied Chemistry and Biochemistry, Faculty of Applied Sciences, University for Development Studies, Navrongo, Ghana

**Keywords:** carbohydrate, complementary/infant food, sensory, simple sugars, sweetpotato, viscosity

## Abstract

**Background:**

Cereal-based complementary foods from non-malted ingredients form a relatively high viscous porridge. Therefore, excessive dilution, usually with water, is required to reduce the viscosity to be appropriate for infant feeding. The dilution invariably leads to *energy and nutrient thinning*, that is, the reduction of energy and nutrient densities. Carbohydrate is the major constituent of food that significantly influences viscosity when heated in water.

**Objectives:**

To compare the sweetpotato-based complementary foods (extrusion-cooked ComFa, roller-dried ComFa, and oven-toasted ComFa) and enriched Weanimix (maize-based formulation) regarding their 1) carbohydrate composition, 2) viscosity and water solubility index (WSI), and 3) sensory acceptance evaluated by sub-Sahara African women as model caregivers.

**Methods:**

The level of simple sugars/carbohydrates was analysed by spectrophotometry, total dietary fibre by enzymatic-gravimetric method, and total carbohydrate and starch levels estimated by calculation. A Rapid Visco™ Analyser was used to measure viscosity. WSI was determined gravimetrically. A consumer sensory evaluation was used to evaluate the product acceptance of the roller-dried ComFa, oven-toasted ComFa, and enriched Weanimix.

**Results:**

The sweetpotato-based complementary foods were, on average, significantly higher in maltose, sucrose, free glucose and fructose, and total dietary fibre, but they were markedly lower in starch content compared with the levels in the enriched Weanimix. Consequently, the sweetpotato-based complementary foods had relatively low apparent viscosity, and high WSI, than that of enriched Weanimix. The scores of sensory liking given by the caregivers were highest for the roller-dried ComFa, followed by the oven-toasted ComFa, and, finally, the enriched Weanimix.

**Conclusion:**

The sweetpotato-based formulations have significant advantages as complementary food due to the high level of endogenous sugars and low starch content that reduce the viscosity, increase the solubility, impart desirable sensory characteristics, and potentially avoid excessive *energy and nutrient thinning*.

The physical and sensory properties of plant-based complementary foods for infants are indirectly of nutritional significance as they impact on the quantity of food eaten and, invariably, nutrient intake. The estimated carbohydrate content of complementary food is between 60 and 75 g/100 g (1) and, thus, is the major factor that influences the viscosity of cooked products (2). When starch slurry is heated, the starch granules swell and increase the viscosity, which directly relates to the quantity of starch (2, 3). Complementary foods that form a very viscous paste during cooking will require excessive dilution with water before they become suitable for feeding infants (3, 4). However, diluting the paste to reduce the viscosity leads to *energy and nutrient thinning* (that is, the reduction of energy and nutrient densities). Therefore, a household-level sweetpotato-based complementary food has been suggested as a more suitable complementary food as it would be lower in starch resulting in a less viscous porridge compared with a maize-based infant product (5).

Apart from viscosity, water solubility index (WSI), which is a measure of the soluble constituents in foods, is another physical property that relates to the carbohydrate composition of foods (6). High WSI can be used to predict the ease of digestion of complementary foods by infants (7, 8), although the actual digestibility in infants was not investigated. Starch is efficiently digested by infants when present in complementary food in small quantities (9, 10); therefore, a higher level of carbohydrate as sugars in complementary foods could be used as an indicator of food digestibility in infants (1, 11).

The level of simple carbohydrates/sugars can affect not only the physical properties (viscosity or solubility) but also the sensory properties and, consequently, food intake. Other researchers reported that infants consumed more cereal-based porridge when sweetened than unsweetened (12).

In an effort to improve the nutritional status of infants in lower-income countries of sub-Saharan Africa, sweetpotato-based complementary foods (extrusion-cooked ComFa, roller-dried ComFa, and oven-toasted ComFa) were proposed as alternatives to commonly used cereal–legume blends, such as Weanimix (13). Weanimix was developed in 1987 in Ghana through collaboration between UNICEF and the Nutrition Unit of the Ministry of Health, Ghana; it contains maize, groundnut, and soyabean or cowpea (14, 15), and it is an improved complementary food compared with traditional cereal-only porridge in energy and protein (15). In our studies, Weanimix was slightly modified by using dehulled maize and soyabean instead of using non-dehulled ingredients, and denoted as enriched Weanimix (5, 16, 17). The extrusion-cooked ComFa and roller-dried ComFa contained 83% of the protein content of 15 g/100 g recommended by the Codex Alimentarius Commission (1), but the oven-toasted ComFa and enriched Weanimix met the protein specification. The ComFa formulations as well as enriched Weanimix met the energy- (1670 kJ/100 g) and fat-stipulated (10–25 g/100 g) levels in the Codex standard (1). However, the sweetpotato-based infant foods have nutritional advantages over enriched Weanimix because of their relatively low phytate, approximately a quarter of the 0.80 g/100 g in enriched Weanimix (16), and high vitamin A (28 vs. 2 µg retinol equivalents/100 kcal) (5). In addition, using the phytate: mineral ratio for predicting calcium, iron, and zinc availability from food, the roller-dried ComFa and oven-toasted ComFa were predicted not to adversely affect the absorption of these essential nutrients as much as the enriched Weanimix (17).

Sucrose levels in sweetpotato roots increases during storage (18–20), and maltose increases when the roots are heated at 65°C or above (18, 20–22). Therefore, it is expected that sweetpotato-based complementary foods would contain higher amounts of simple sugars and lower starch as previously suggested (5). Consequently, the sweetpotato-based infant foods would be expected to have lower viscosity and be more soluble in water and, thus, require less dilution to reach an acceptable viscosity for infant feeding, that is, less *energy and nutrient thinning*.

The objectives of this study were to compare the sweetpotato-based complementary foods (extrusion-cooked ComFa, roller-dried ComFa, and oven-toasted ComFa) and enriched Weanimix (maize-based formulation) regarding their 1) carbohydrate composition, 2) viscosity and WSI, and 3) sensory acceptance evaluated by sub-Sahara African women as model caregivers.

## Materials and methods

### Processing of complementary food formulations

The choice and the proportion of the ingredients and the processing of the sweetpotato-based infant foods (ComFa formulations) have been previously reported (13, 16). The complementary foods were formulated to meet the recommended macronutrients specification of the Codex standard (1), and for easy replication at both the household- and industrial-level as previously discussed (13). Briefly, the sweetpotato-based formulations were processed as follows.

### Extrusion-cooked ComFa and roller-dried ComFa (Industrial-level)

Cream-fleshed sweetpotato flour (72%), soyabean flour (15%), soyabean oil (6.0%), iodised salt (0.50%), sugar (0.50%), and skimmed milk powder (6.0%) were mixed, and the composite flour was either extrusion cooked or roller dried. Extrusion cooking of the composite flour was done at 120°C. For the roller-dried ComFa, slurry was prepared, cooked in a steam-jacketed pan for 10 min at 80–83°C before roller drying with steam at 107°C under 100 kPa.

These are the industrial-level ComFa formulations, which could be reconstituted in the same way as Nestlé Cerelac^®^, a popular proprietary dry infant cereal-based food in Africa.

### Oven-toasted ComFa (Household-level)

Composite flour of sweetpotato (66%), soyabean (10%), soyabean oil (6.0%), iodised salt (0.50%) sugar (0.50%), and fish powder from anchovy without the heads (17%) was toasted in a 120°C pre-heated oven for 30 min. This is the household-level sweetpotato-based formulation.

### Enriched Weanimix

A maize-based complementary food, described as Weanimix, was processed as previously described (14, 15), with a slight modification (5) by further enrichment with 17% (wt/wt) anchovy powder and 0.50% (wt/wt) sugar.

### Chemical and physical analyses

#### Carbohydrate composition

Three samples of each complementary food were separately defatted using the Soxhlet method (23), and then oven-dried at about 108°C for 16 h to a constant weight. An assay kit (K-MASUG 10/04, Megazyme Int., Wicklow, Ireland) was used to determine the levels of simple carbohydrates/sugars: maltose, sucrose, and free glucose (the assay enzymes do not hydrolyse glucose from glucose polymers) in the defatted samples using a UV/Visible spectrophotometer (Pharmacia LKB Ultrospec II, England) at 340 nm. Approximately 0.5 g of each defatted sample was weighed and transferred into 50-mL beakers and deproteinised as described in the kit to reduce possible interference by other substances.

Total available carbohydrate was calculated as 100% minus the sum of moisture, protein, fat, ash, and total dietary fibre obtained using proximate analysis as previously reported (16). Levels of free fructose and starch contents in the samples were estimated by calculation, while total dietary fibre in the samples was analysed by enzymatic–gravimetric method using the K-ACHDF 11/08 kit (Megazyme Int., Wicklow, Ireland). Free fructose was calculated by adjusting for the fructose from the sugar used as an ingredient after determining the total fructose levels in the formulations using the K-ACHDF 11/08 assay kit by spectrophotometry at 340 nm. The starch content was calculated as the difference between total available carbohydrate and the sum of maltose, sucrose, free glucose, and free fructose; corrected for approximately 3 g/100 g of lactose from skimmed milk powder (24) used as an ingredient in processing the extrusion-cooked ComFa and roller-dried ComFa.

### Viscosity measurement

A Rapid Visco Analyser (RVA) [Super 4 (RVA-S4), Newport Scientific Pty Ltd., NSW, Australia], interfaced with a personal computer equipped with the software Thermocline for Windows v3.0, was used to measure the pasting profile using the Newport Scientific Method Version 13. In summary, about 3 g (14% moisture basis) of non-defatted complementary foods (in triplicate) were weighed and transferred to aluminium canisters containing approximately 25 mL of distilled water (corrected to compensate for 14% moisture used for the sample). A plastic paddle was used to mix the slurry to break up any lumps before inserting the canister with the paddle into the instrument to measure the pasting profile for 20 min. The initial equilibration of the slurry was set for 2 min at 25°C, which was followed by heating to a maximum of 95°C for 5 min, and then holding at this temperature for a further 3 min. The paste was cooled to 25°C for 5 min, and held at this temperature for 3 min.

The maximum apparent viscosity during heating, or the heating/holding phase of the test, was reported as the peak viscosity, and the final viscosity was the viscosity recorded at the end of the test (25). Using the recommended viscosity measurement at 45°C for infant porridge (12, 26), the average of seven apparent viscosity readings between 13.60 and 14.00 min (RVA time) corresponding to 45°C was taken as *consume viscosity*.

### Water solubility index

The gravimetric method described by Jin et al. (27) was used to determine the WSI of the non-defatted infant formulations in triplicate.

### Consumer sensory evaluation

The sensory acceptance of the products, roller-dried ComFa, oven-toasted ComFa, and enriched Weanimix, were measured using the method of Stone and Sidel (28). To reduce evaluation fatigue, the extrusion-cooked ComFa was not included for the sensory acceptance test. Twenty women from nine sub-Saharan Africa countries (Congo, Gabon, Ghana, Kenya, Malawi, Nigeria, Rwanda, Zambia, and Zimbabwe), resident in Palmerston North (PN), New Zealand (NZ), who had experience of feeding a child with cereal-based porridge prepared at the household level and responded to the advertisement to participate in the consumer sensory evaluation were invited to the Institute of Food, Nutrition and Human Health, Massey University, PN, NZ. Mothers have been employed to assess the consumer acceptance of cassava–soyabean complementary foods (29). The sensory attributes evaluated for the selected complementary foods included overall acceptability, colour, smell, texture, and taste using a 9-point hedonic scale with 1 as least acceptable/dislike extremely and 9 as highly acceptable/like extremely. A just-about-right scale (1 = much too low, 5 = just-about-right, and 9 = much too high) was used to evaluate the level of sweetness and saltiness. The women were also asked whether they would be willing to feed their babies with each of the complementary food formulations using a 9-point scale (1 = not likely; 5 = neutral; 9 = very likely).

Ethical approval was obtained from the Massey University Human Ethics Committee (HEC: Southern A Application-09/58), PN, NZ. Before the sensory test began, written consent to take part in the study was obtained from each participant after explaining the objectives to her.

Two of the participants who were feeding complementary foods to their babies at the time of the sensory evaluation testing were selected to prepare suitable food for babies from the roller-dried ComFa, oven-toasted ComFa, and enriched Weanimix, as they would do at their homes in sub-Saharan Africa. A total of 150 g of each of the three formulations were given to the selected women. These two women were informed that oven-toasted ComFa and enriched Weanimix were to be prepared as traditional household-level infant porridge while the roller-dried ComFa was to be reconstituted in the same way as Nestlé Cerelac.

For sensory testing, approximately 20 g of each of the prepared samples were randomly served together to all of the participants (including the two caregivers who prepared the porridge) by the researchers in three-figure-coded plastic cups at room temperature for the product liking evaluation in a sensory booth.

### Statistical analysis

The composition of carbohydrate, viscosity, and the WSI data were subjected to Univariate Analysis of Variance (one-way ANOVA). The Tukey's studentised range test was used to compare differences between means when the ANOVA was significant (*P* <0.05). The relationship between apparent viscosity and WSI and either the sum of simple sugars, starch, or total dietary fibre was evaluated using Pearson's correlation. Minitab v15.1™ (Minitab Inc., State College, PA, USA) was used for the statistical analyses above.

The sensory acceptance test data were statistically analysed using both the Kruskal-Wallis non-parametric test (not assuming normality of data) for three or more independent samples and the usual one-way ANOVA, assuming normality of data using the PASW Statistics 18, Release Version 18.0.0 (SPSS, Inc., 2009, Chicago, IL, USA).

For the sweetness and saltiness intensities scoring using the just-about-right scale, the responses were categorised as ‘much too low’ (≤2), ‘too low’ (3, 4), just-about-right (5), ‘too high’ (6, 7), and ‘much too high’ (≥8). As recommended by Stone and Sidel (28), if 70% of the responses were in the just-about-right category, the intensity was taken as acceptable by the caregivers.

## Results

The levels of sugars and starch in all of the infant foods are presented in Table 1. As expected, the sum of simple sugars as a percentage of total carbohydrate (that is, total dietary fibre and total available carbohydrate) was approximately 64% for the ComFa formulations but only about 6.0% for the maize-based formulation. There were significant differences in the levels of simple carbohydrates between the sweetpotato-based formulations compared with those of the maize-based product. On average, sweetpotato-based formulations were higher in maltose (26 times, *P* <0.0001), sucrose (five times, *P* <0.0001), free glucose (19 times, *P* <0.0001), and fructose (7 times, *P*=0.002). The roller-dried ComFa was slightly higher in maltose than the extrusion-cooked ComFa (1.1 times) and oven-toasted ComFa (1.2 times). All the other carbohydrate fractions, with the exception of free glucose, were not significantly different between the ComFa formulations. The sum of maltose, sucrose, and glucose in the sweetpotato-based complementary foods is comparable to the level of 50 g/100 g (dry matter basis) in sweetpotato roots reported by other researchers (18). The sweetpotato-based complementary foods contained significantly less starch (10–13 vs. 47 g/100 g, *P* <0.0001), and they were higher in total dietary fibre (8–11 vs. 6 g/100 g) than the levels in enriched Weanimix. The levels of the calculated total available carbohydrate and total dietary fibre together in the ComFa formulations indicated that the sweetpotato-based formulations satisfied the estimated carbohydrate content of 60–75 g/100 g in the Codex standard (1), but the enriched Weanimix was slightly lower by about 6.0%.

**Table 1:** Carbohydrate composition^1^ (g/100 g dry matter basis) of sweetpotato- and maize-based complementary foods

Complementary food	Maltose	Sucrose	Free glucose	Free fructose	Starch^2^	Total dietary fibre^3^	Total available carbohydrate^4^
Sweetpotato-based							
Extrusion-cooked ComFa	27.50 (2.45)^a^	10.20 (0.31)^a^	1.24 (0.04)^b^	3.07 (0.37)^a^	11.32 (1.87)^b^	10.25 (2.29)^a^	56.07 (1.97)^a^
Roller-dried ComFa	30.85 (3.84)^a^	10.53 (1.06)^a^	1.34 (0.03)^a^	2.94 (1.04)^a^	10.53 (3.70)^b^	10.57 (1.21)^a^	58.92 (0.83)^a^
Oven-toasted ComFa	25.43 (1.17)^a^	10.08 (0.43)^a^	1.40 (0.05)^a^	2.61 (0.46)^a^	13.75 (0.72)^b^	8.16 (0.77)^a,b^	53.28 (0.94)^a,b^
Maize-based							
Enriched Weanimix	1.06 (0.18)^b^	2.01 (0.12)^b^	0.07 (0.02)^c^	0.39 (0.13)^b^	46.72 (0.64)^a^	6.08 (0.44)^b^	50.25 (0.75)^b^
*P*	<0.0001	<0.0001	<0.0001	0.002	<0.0001	0.01	<0.0001

1 Value is mean (standard deviation) of triplicate; values with the same superscript letter in a column are not significantly different (*P*>0.05).

2 Starch = total available carbohydrate minus sum of maltose, sucrose, free glucose, and free fructose; the starch content for extrusion-cooked ComFa and roller-dried ComFa was corrected for approximately 3 g/100 g of lactose from skimmed milk powder (24), an ingredient.

3,4 Data previously published (16); Tukey's studentised range test was used for the post-hoc mean analysis instead of least significant difference in published manuscript.

**Fig. 1:**
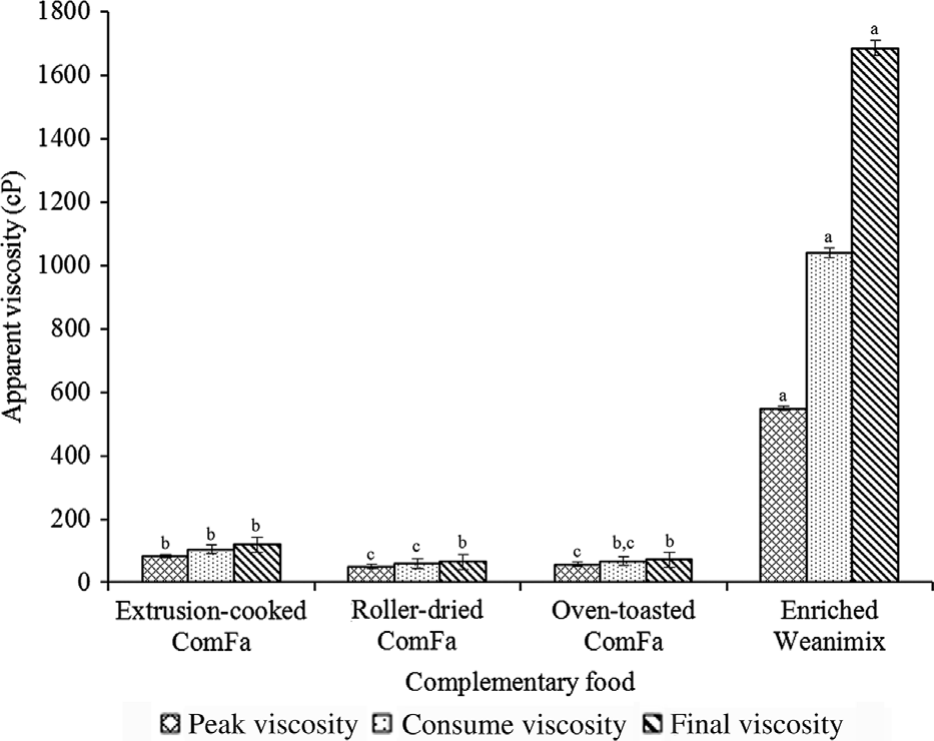
Viscosity during cooking (peak viscosity), at serving (consume viscosity), and during storage (final viscosity) of porridge from the complementary foods. Bar value (mean±standard deviation, *n*=3). Bar with different letter for each variable for complementary foods was significantly different (*P*<0.0001**)**.

The average apparent viscosity of the sweetpotato-based infant foods was 9-, 13-, and 20-times lower at the peak, *consume* and final viscosities compared with enriched Weanimix (Fig. 1). All the complementary foods showed an increase in viscosity from high temperature (peak viscosity) to low temperature (final/storage viscosity). The increase in viscosity was less pronounced (1.4-fold) in the sweetpotato-based products but substantially high (3.1-fold) for the maize-based formulation. The differences in apparent viscosities were not significant for extrusion cooked ComFa.

**Fig. 2:**
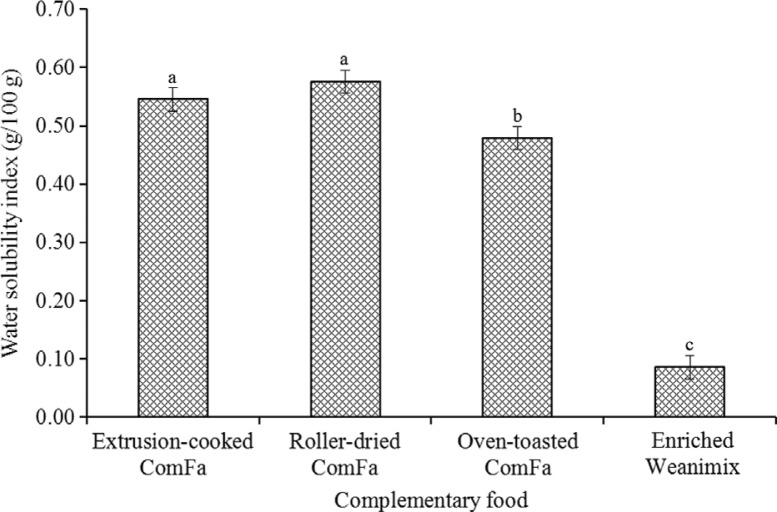
The water solubility index of the complementary foods. Bar value (mean±standard deviation, *n*=3). Bar with a different letter for each variable was significantly different (*P*<0.0001).

The ComFa formulations were significantly higher in WSI (6.6 times, *P* <0.001) compared with the maize-based complementary food (Fig. 2).

The relationships between the simple carbohydrates, total dietary fibre as well as starch and the physical properties (viscosity and solubility) are shown in Table 2. As expected, the level of simple sugars correlated negatively with the viscosities and positively with WSI.

**Table 2:** Correlation coefficient^1^ between carbohydrate composition, apparent viscosity, and water solubility index (WSI)

	Physical property			
	
Carbohydrate fraction	Peak viscosity^2^	Consume viscosity^3^	Final viscosity^4^	WSI
Simple sugars^5^	−0.992 (0.008)	−0.992 (0.008)	−0.992 (0.008)	0.998 (0.002)
Total dietary fibre	−0.852 (0.148)	−0.854 (0.146)	−0.854 (0.146)	0.935 (0.065)
Starch	0.995 (0.005)	0.996 (0.004)	0.996 (0.004)	−0.995 (0.005)

1 Cell content: Pearson correlation coefficient (*P*-value) using all the formulations.

2 Maximum apparent viscosity during heating or the heating/holding phase during Rapid Visco Analyser (RVA) test.

3 Viscosity corresponding to approximately 45°C.

4 Viscosity during storage, recorded at 25°C.

5 Sum of maltose, sucrose, free glucose, and fructose.

A slurry oven-toasted ComFa and enriched Weanimix was prepared with potable water before cooking on a hot-plate for about 10 min. The roller-dried ComFa was reconstituted as ready-to-eat complementary food using potable water that was boiled and allowed to cool to 40°C. The average age of the participants who took part in the consumer sensory evaluation was 37±8 year (mean±standard deviation). All of the women also indicated that sweetpotato was readily available in their respective countries. The oven-toasted ComFa was mixed with approximately 750 mL of potable water before cooking, but the enriched Weanimix was initially mixed with about 600 mL of water, with a further addition of about 300 and 150 mL of water during cooking to reduce the viscosity. Approximately 450 mL of the warm water was used for reconstitution of the roller-dried ComFa. The amount of water used for the reconstitution was discussed and accepted by the two selected women, and the final viscosity was unanimously accepted by the other participants.

**Fig. 3:**
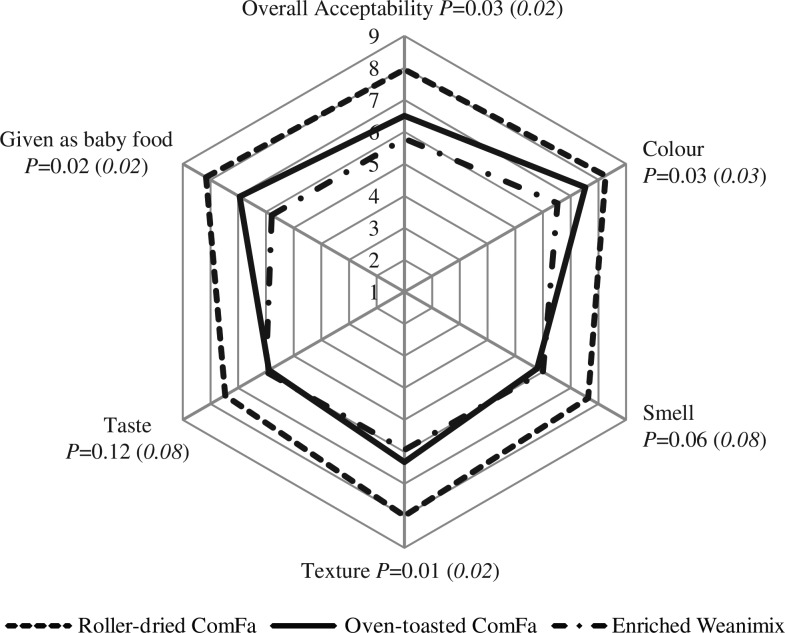
Diagrammatic presentation of product liking for sensory attributes and choice to give the formulations as complementary food to infants by 20 female sensory participants. Sensory attribute with *P*<0.05 indicates that significant difference among the complementary foods; italicised value in parenthesis is asymptotic significance from Kruskal–Wallis test. A 9-point hedonic scale was used (1 = least acceptable/dislike extremely; 5 = neutral; 9 = highly acceptable/like extremely) for all attributes except for willingness to give product to babies (1 = not likely; 5 = neutral; 9 = very likely).

Both the Kruskal–Wallis non-parametric test and the usual ANOVA gave similar results regarding the rejection of the null hypothesis (Fig. 3). Therefore, ANOVA was chosen, as recommended by Montgomery (30), to compare the means of the scores of the sensory attributes evaluated by the participants. The order of liking among the products was roller-dried ComFa, followed by oven-toasted ComFa, and, finally, enriched Weanimix for colour, texture, overall acceptability, and willingness to use the formulations in their households as complementary food (Fig. 3). All of the three formulations were judged to be acceptable on all of the indicators. The mean scores of sensory liking for colour, texture, overall acceptability, and willingness to give the product as baby food on the 9-point scale ranged from 6.3 to 8.3 for the two ComFa formulations selected for the sensory study. In contrast to the ComFa formulations, the equivalent range for the above sensory attributes was 5.8 to 6.5 for the enriched Weanimix.

**Fig. 4:**
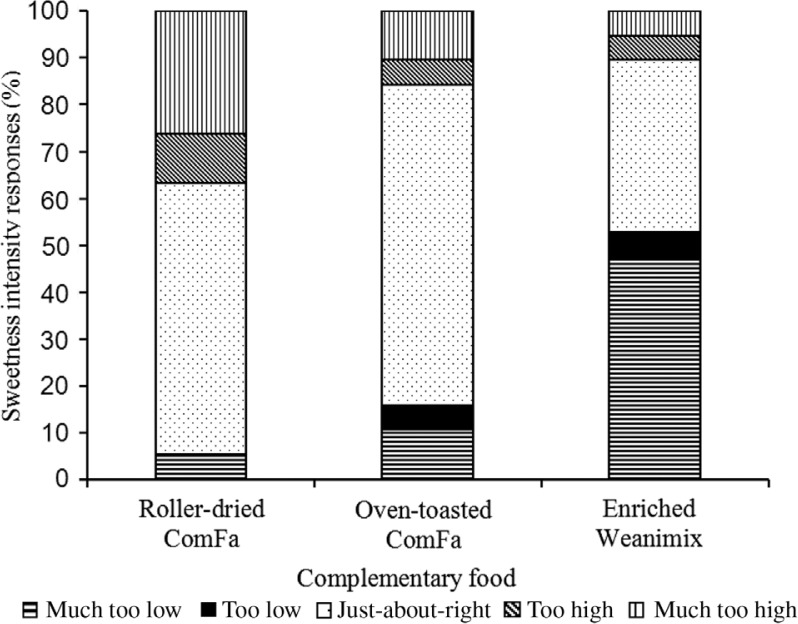
Diagrammatic presentation scoring for sweetness intensity of the complementary food formulations on just-about-right scale by 20 female sensory participants. Scoring was done on a 9-point scale, and responses were categorised as ‘much too low’ (≤2), ‘too low’ (3, 4), just-about-right (5), ‘too high’ (6, 7), and ‘much too high’ (≥8).

**Fig. 5:**
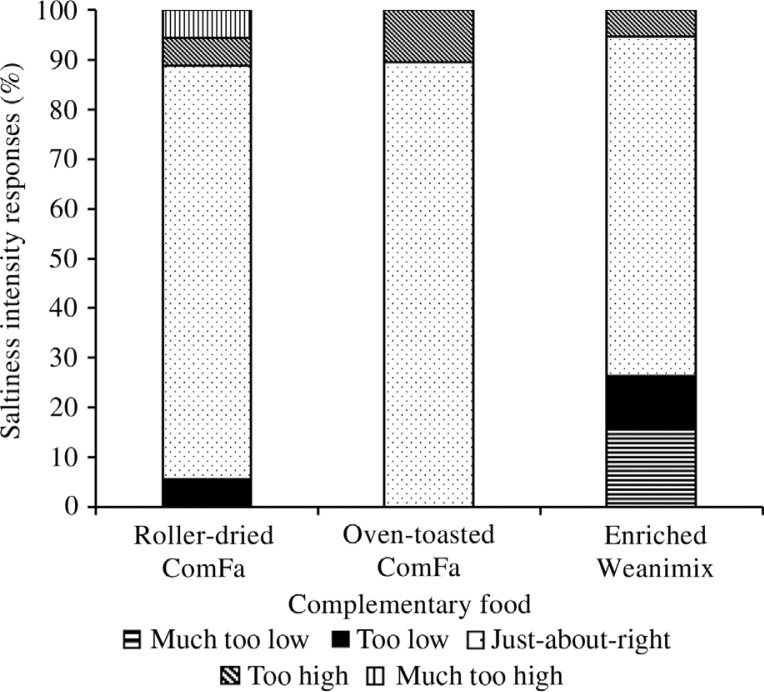
Diagrammatic presentation scoring for saltiness intensity of the complementary food formulations on just-about-right scale by 20 female sensory participants. Scoring was done on a 9-point scale, and responses were categorised as ‘much too low’ (≤2), ‘too low’ (3, 4), just-about-right (5), ‘too high’ (6, 7), and ‘much too high’ (≥8).

For analysing the perceptions for sweetness (Fig. 4), about 58 and 68% of the caregivers regarded the intensity of sweetness as just-about-right for roller-dried ComFa and oven-toasted ComFa, respectively, compared with 37% for enriched Weanimix. About 53% of the caregivers scored the sweetness intensity of enriched Weanimix as being at least too low. However, the percentage of the score in the just-about-right category for all the formulations did not met the established agreed-on acceptability of 70%. Most of the respondents, over the established agreed-on acceptability 70%, regarded the intensity of the saltiness just-about-right for both ComFa-products (Fig. 5). For Weanimix, almost quarter of the respondents regarded its saltiness as too low.

## Discussion

The higher level of sugars in sweetpotato-based complementary foods will make them naturally sweeter than the maize-based product. Therefore, the addition of sugar would not be necessary when preparing porridge. The natural sweetness of the sweetpotato-based infant foods could offer a nutritional benefit over the maize-based complementary food as it may lead to a higher intake of food (12).

The slightly higher level of maltose in the roller-dried ComFa than the extrusion-cooked ComFa and oven-toasted ComFa is likely due to gradual heating of the slurry that allows the endogenous β-amylase in sweetpotato (22) a longer interaction time with gelatinised starch before being denatured. However, for the extrusion-cooked and oven-toasted ComFa, the composite flour was introduced into the extruder or the oven, both pre-heated to 120°C, which could lead to early denaturation of β-amylase. A similar trend was observed when the roots of cream-fleshed sweetpotato, the same variety used in this study, were mashed and heated slowly from 75 to 100°C for 40 min compared with those cooked rapidly for 10 min at either 95–100°C or 105°C (20).

The total dietary fibre in the ComFa formulations exceeded the maximum specified content of 5 g/100 g in the Codex standard (1). As discussed in an earlier publication (16), the high levels of fibre in the sweetpotato-based formulation may be beneficial rather than a nutritional limitation. It has been reported that approximately 25–50% of the fibre in sweetpotato is soluble (31, 32), which may serve as fermentable substrate for health-promoting colonic bacteria.

The peak viscosity measured during heating, or the heating/holding phase on the RVA, represents the maximum viscosity likely to occur during porridge preparation on a kitchen stove. For example, in Ghana, slurry is made from cereal or cereal-based dough/flour in a cooking pot, which is then cooked on a stove with frequent stirring. The porridge is usually left to cool to between 40 and 45°C before being fed to infants. The apparent viscosity at approximately 45°C (the temperature of porridge served to infants), thus represents the likely consistency of the porridge given to infants (4, 12, 26). The final viscosity, measured at 25°C, could correspond to the storage viscosity of the porridge when left to cool at room temperature. The increase in apparent viscosity upon cooling observed in this study compares well with similar observation in other studies (4, 33), and may be due to the partial association or realignment of starch granules (particularly, the linear molecules, amylose, and linear parts of amylopectin molecules) to form a precipitate or a gel, a phenomenon referred to as retrogradation (2). The substantial increase in the viscosity during cooling in the enriched Weanimix indicates higher retrogradation, and it can be related to the corresponding higher starch content than the ComFa formulations. The suggestion in our earlier publication that the oven-toasted ComFa will be lower in starch and, consequently, have a lower viscosity compared with the enriched Weanimix, both as household-level complementary food (5), is confirmed in this study.

The higher level of sugars and the lower starch content in the ComFa formulations partly contributed to the lower apparent viscosities of the sweetpotato-based products as reported in other studies (34, 35). Because of the inherently low viscosity of the sweetpotato-based formulations, less water will be added during porridge preparation than would be needed for the maize-based formulation so there will be less *energy and nutrient thinning* for the ComFa formulation.

The high WSI of the sweetpotato-based complementary food in this study can be explained by the quantity of soluble molecules (e.g. sugars and possibly soluble fibre). The relatively high WSI and simple carbohydrates to starch ratio may suggest easier digestibility of the sweetpotato-based formulations than enriched Weanimix as proposed by other researchers (7–9). The lower solubility of the oven-toasted ComFa compared with the other ComFa formulation could be due to the slightly higher starch content.

The participants in the sensory evaluation test were purposely recruited because the ComFa formulations were developed for infants in sub-Saharan Africa. The women were chosen instead of infants because of the common practice (e.g. in Ghana), that caregivers taste food before serving to their babies, hence they could serve as suitable consumer sensory panellists for complementary foods as done in another study (29). Our sampling criteria limited us from achieving the recommended 25–50 responses per product for reliable and credible laboratory-based sensory liking test (28). In any case, the reasons for the number of responses per product include improving the statistical power for detecting minimal differences in the hedonic scale ratings given for products (28). Notwithstanding this limitation, there was a clear significant trend (*P* <0.05) that indicated that the sweetpotato-based complementary foods were preferred by the participants above? the maize-based product for all the eight attributes evaluated, including the willingness to give the products as baby food. The just-about-right sweetness intensity score for the ComFa formulations supports our earlier suggestion that mothers would probably not sweeten the porridge for infants with sucrose. The higher consumer rating for the roller-dried ComFa compared with the oven-toasted ComFa was probably because the fish powder included in the oven-toasted ComFa imparted a flavour that was disliked by some of the participants. We have previously discussed elsewhere that the fish powder was incorporated into the household-level formulation to improve the protein quality and calcium (5).

Another important observation of nutritional significance relates to the amount of water used to prepare the same quantities of complementary food flour. Less water was used for the oven-toasted ComFa porridge compared with the enriched-Weanimix [1:5 (wt/wt) vs. 1:7 of flour: water, respectively]. Therefore, it is the desired viscosity of porridge that guides mothers/caregivers on the suitable proportions of complementary food flour and water to be used during preparation. The lower amount of water used for the oven-toasted ComFa porridge indicates less *energy and nutrient thinning* compared with the maize-based formulation porridge. Also, the amount of water used for the maize-based porridge supports the relatively high peak viscosity of this product.

The sweetness score and the amount of water used for the porridge correspond to the data on the composition of carbohydrate and apparent viscosity, respectively. This therefore validates the sensory data.

## Conclusion

The sweetpotato-based formulations had a higher sugar to starch ratio than the maize-based complementary food. The levels of the total sugars and starch are the likely reasons for the lower apparent viscosity and higher WSI of the ComFa formulations compared with the enriched Weanimix resulting in requirement for less water when preparing the porridge from the ComFa formulations. The scores of the consumer acceptance given by the infant caregivers indicated highest liking for roller-dried ComFa, followed by the oven-toasted ComFa, and, finally, the enriched Weanimix. These findings, combined with the relatively low phytate and phytate: mineral ratio and high vitamin A levels (5, 16, 17), suggest that the use of sweetpotato in complementary food should be encouraged as it has potential to have significant nutritional benefit in low-income countries.
